# Adherence to and acceptability of artemether-lumefantrine as first-line anti-malarial treatment: evidence from a rural community in Tanzania

**DOI:** 10.1186/1475-2875-9-48

**Published:** 2010-02-11

**Authors:** Abdunoor M Kabanywanyi, Christian Lengeler, Prudensiana Kasim, Said King'eng'ena, Raymond Schlienger, Nathan Mulure, Blaise Genton

**Affiliations:** 1 Ifakara Health Institute, P.O. Box 78373, Kiko Avenue, Old Bagamoyo Road, Mikocheni, Dar es Salaam, Tanzania; 2 Swiss Tropical Institute, Socinstrasse 57, Basel, CH-4056, Switzerland; 3 Novartis Pharma AG, Lichtstrasse 35, Basel, CH-4056, Switzerland; 4 Novartis Pharma (EACA), Hospital Road, Upper Hill, Nairobi, P.O. Box 46057 - 00100 GPO, Kenya

## Abstract

**Background:**

Controlled clinical trials have shown that a six-dose regimen of artemether-lumefantrine (AL) therapy for uncomplicated *Plasmodium falciparum *malaria results in cure rates >95% with good tolerability.

**Materials and methods:**

A prospective study was carried out to document the adherence to and acceptability of AL administration. This was undertaken in the context of the ALIVE study, a prospective, community-based, observational study in a rural, malaria-endemic area of Tanzania. Following microscopic confirmation of *P. falciparum *infection, the first AL dose was taken under supervision, with the subsequent five doses taken unsupervised at home. Patients were randomized to receive a home-based assessment close to the scheduled time for one of the unsupervised doses, but were blinded to which follow-up visit they had been allocated. A structured questionnaire was administered by trained staff and AL consumption was confirmed by inspection of blister packs.

**Results:**

A total of 552 patients were recruited of whom 352 (63.8%) were <13 years old. The randomization process allocated 112, 109, 110, 100 and 111 patients to a follow-up visit after doses 2, 3, 4, 5 and 6, respectively. For dose 2, 92.0% of patients (103/112) correctly took AL at 8 ± 1 hours after dose 1. The remaining doses were taken within four hours of the correct time in 87-95% of cases. Nine patients (1.7%) missed one dose. Blister packs were available for inspection in 548 of cases (99.3%) and confirmed patient-reported data that the previous dose had been administered. Nearly all patients took AL with water (549/552 [99.5%]). Two patients (0.4%) took the drug with food. The dosing pictogram and clustering of tablets within the blister packs was considered helpful by 91.8% and 100.0% of patients, respectively. Overall, 87.1% of patients (481/552) found AL easier to take/administer than sulphadoxine-pyrimethamine (SP) and 87.7% (484/552) believed that AL was more effective than SP.

**Discussion:**

Factors contributing to adherence were likely to be helpful packaging, pictorial dosing instructions and patients' conviction that AL is effective.

**Conclusion:**

Adherence to the dosing regimen and timing of AL administration was very good.

## Background

Malaria is the leading cause of outpatient and inpatient admissions in most sub-Saharan African countries, including Tanzania, and continues to exert a high burden in terms of mortality [1-3], morbidity [1,4] and health expenditure [5,6]. The use of anti-malarial drugs for chemotherapy and chemoprophylaxis is a critical component in the fight against malaria, but *Plasmodium falciparum *resistance to conventional anti-malarials, such as chloroquine and sulphadoxine-pyrimethamine (SP), is high [1,7-10]. It is for this reason that the World Health Organization (WHO) has been recommending artemisinin-based combination therapy (ACT) as first-line therapy to replace failing anti-malarial drugs [11].

The ACT artemether-lumefantrine (AL, Coartem^®^) combines the short-acting artemisinin derivative artemether with long-acting lumefantrine. Giving the second dose of AL eight hours after the first dose quickly achieves and maintains the blood concentration of artemether above the minimum effective concentration [12] to help ensure that malaria parasites are exposed to high levels during the middle third of their life cycle, when they are most susceptible to anti-malarial agents [13]. AL has demonstrated a high level of efficacy and a good tolerability profile [14-18]. As a result, the national treatment policy of Tanzania was revised in November 2006 to adopt AL as first-line treatment for uncomplicated malaria.

AL is administered as a six-dose regimen over a period of three days [19]. Controlled trials using this regimen have demonstrated cure rates of over 95% [20-23], consistent with recommendations from WHO that cure rates for uncomplicated *P. falciparum *malaria should be at least 90% and preferably exceed 95% [11]. Evidence regarding the adherence to the AL dosing regimen and feasibility of its use in programmatic conditions remains limited [24-29]. Nevertheless, proper evaluation of adherence and acceptance outside the context of controlled clinical studies is important as AL is widely deployed throughout malaria endemic sub-Saharan countries.

Following the inclusion of AL within the national Tanzanian treatment policy, the ALIVE ('artemether-lumefantrine in vulnerable patients: exploring health impact') study was undertaken in a rural, malaria-endemic area of the country with the aim of evaluating the impact of introducing AL as first-line malaria treatment on malaria-related morbidity and mortality. As part of ALIVE, a prospective study was conducted under routine conditions to document the adherence to and acceptability of AL drug administration in this setting.

## Methods

### The ALIVE study

ALIVE is a prospective, observational, community-based, longitudinal, demographic surveillance study taking place in two rural districts of Tanzania (Ulanga and Kilombero). The primary objective is to assess the effect of AL on all-cause mortality in infants and children ≥3 months of age (and >5 kg) and <5 years old, using historical data based on the former first-line treatment with SP as comparator. Secondary objectives include assessment of adherence to the AL regimen, knowledge of correct AL intake and patient satisfaction.

The conduct of the ALIVE study complies with the Declaration of Helsinki. The study protocol was approved by the institutional review board of the Ifakara Health Institute (IHI), which implements the ALIVE study, and the Tanzanian National Institute for Medical Research (NIMR).

### Administration of AL

As per the instructions from the manufacturer (Novartis Pharma AG, Basel, Switzerland), AL is dosed according to body weight: 5 to <15 kg, one tablet per dose; 15 to <25 kg, two tablets per dose; 25 to <35 kg, three tablets per dose; ≥35 kg, four tablets per dose. The first two doses of AL are to be given eight hours apart on day 1. On days 2 and 3, AL is to be given twice daily, 12 hours apart; with the morning dose being administered 24 hours after the first dose was taken.

In this study, the first dose of AL was taken under supervision at the health facility, with the subsequent five doses taken unsupervised at home. Dosing and time of taking/administering the drugs was explained and the time was clearly marked on the AL blister packs by the dispensing healthcare provider. Patients or caregivers were advised to ensure AL was not taken/given on an empty stomach.

### Assessment of adherence and acceptability

Assessment of adherence to and acceptability of AL was conducted within the context of the ALIVE study and was undertaken at the Mlimba Health Center in the Kilombero District during the period March to April 2008, i.e. approximately one year after the new treatment policy was introduced in Tanzania (January 2007). Patients living in villages no further than six kilometers from the Mlimba Health Center were eligible to take part in the assessment if they had no clinical signs of complicated malaria and *P. falciparum *infection was confirmed by blood smear. Informed consent was obtained from all patients.

A computer-generated randomization list was used to allocate eligible patients to a home visit for one of the five doses of AL to be taken after the initial, supervised dose. It was planned that all patients be visited at home at a time close to the scheduled time of AL administration. In the event that patients were due to be visited late in the evening or at night following an evening dose, visits were scheduled for the following morning. Patients or caregivers were informed that there would be a follow-up visit but were blinded as to which of the five possible follow-up visits they had been allocated.

Each visit was undertaken by one of two IHI field workers or three local field assistants, after appropriate training based on standard AL training materials provided by the drug manufacturer (Novartis) which had been field tested by researchers from IHI in Ifakara. For patients younger than 13 years, the patient's caregiver was interviewed instead of the patient.

### Questionnaire

A structured questionnaire (see Additional file 1) was developed by researchers from IHI and the Swiss Tropical Institute and Novartis, after which it was field tested by researchers from IHI in Ifakara, and then administered to the patient or caregiver at each visit. This included questions on the number of doses to be administered, the number of tablets per dose, the exact time at which the last dose was given, reason for any missed doses, appropriate action if a dose was vomited, consumption of concomitant food or drink at the time of AL dosing, and how patients/caregivers remembered that AL doses should be taken. Additional questions included whether the instructions/drawing on the AL pack were considered useful, how patients/caregivers perceived the clustered doses in the blister packs, how easy it was for them to take/administer AL, how effective they judged AL as compared to SP (the previous first-line therapy for uncomplicated malaria in Tanzania), and their preference for AL over other treatments (antibiotics, analgesics/antipyretics, quinine injection, herbs from traditional healer or remedy from witchdoctor). Consumption of the dose was confirmed by inspection of the AL blister packs.

## Results

### Patient population

A total of 552 patients met the eligibility criteria and were recruited for the study (Table 1). The majority of patients (352/552 [63.8%]) were aged less than 13 years. All included patients had come to the medical facility to seek medical attention for fever. Almost all patients (544/552, 98.6%) were reported to be unwell or moderately unwell at the time of presentation to the health facility.

**Table 1:** Patient characteristics (n = 522)

Attribute	N (%)
Age	
<13 years	352 (63.8)
≥13 years	200 (36.2)

Female gender	319 (57.8)

Level of education of patient/caregiver	
None	63 (11.4)
Primary school	421 (76.3)
Secondary school	68 (12.3)
College	0 (0)

Occupation of patient/caregiver	
Employed	22 (4.0)
Self employed	77 (13.9)
Farmer	369 (66.9)
Other	84 (15.2)

Age	
3 months - 3 years	270 (48.9)
3 - 8 years	62 (11.2)
8 - 12 years	39 (7.1)
>12 years	181 (32.8)

Patient condition on presentation at health facility	
Very unwell	0 (0)
Unwell	399 (72.2)
Moderately unwell	145 (26.3)
Moderately well	2 (0.4)
Well	1 (0.2)
Perfectly well	5 (0.9)

The randomization process allocated 112, 109, 110, 100 and 111 patients to a follow-up visit after doses 2, 3, 4, 5 and 6, respectively.

### Adherence to AL

AL was dispensed at the Mlimba Health Center in all cases. When asked, 100% of patients/caregivers reported that they had received an explanation of how to use AL. Results of the questionnaire confirmed that all patients/caregivers understood the number of doses required, and the number of tablets that should be taken/administered per dose (Table 2). All but one patient responded that five doses in total were to be taken, an answer that was correct when referring only to the doses for which they were responsible (the first dose was given by the healthcare provider). AL was taken at the correct time in approximately 90% of cases for each dose (Table 2). For dose 2, 92.0% of patients (103/112) took AL at 8 ± 1 hours after dose 1. The remaining doses were taken within four hours of the correct time in 87-95% of cases. In total, nine out of 522 patients (1.7%) reported missing a single dose of AL. In two out of these nine cases, the patient had forgotten to take the final dose. One patient used the intended dose to replace a dose vomited previously, and one patient ceased to take the drug after vomiting. In the remaining five patients there was no apparent reason for missing prescribed doses. AL blister packs were available for examination at the randomized visit in 548 cases (99.3%), and the reported number of doses taken corresponded with actual pill count at each visit. No patient missed more than one dose, and no patient missed dose 2. In case of a dose being vomited, the majority of patients correctly understood that they should return to the health facility for a replacement dose (316/552, 57.3%). However, a relatively high proportion of patients (42.7%) incorrectly believed that a replacement dose could be taken from the existing blister pack or that no action was required.

**Table 2:** Assessment of adherence to the AL dosing regimen, as evaluated by questionnaire (n = 522)

	N (%)
How many doses in total to be administered for a complete course of treatment	
1	0
2	0
3	0
4	0
5	551(99.8)
6	1 (0.2)

Number of tablets per dose to be taken?	
1 tablet	270/270 (100)
2 tablets	62/62 (100)
3 tablets	40/40 (100)
4 tablets	180/180 (100)

AL dose taken at correct time (i.e. ± 1 hours for dose 2, ± 4 hours for doses 3-6)	
Dose 2	103/112 (92.0)
Dose 3	103/109 (94.5)
Dose 4	100/110 (91.0)
Dose 5	96/110 (87.0)
Dose 6	99/111 (89.2)

Number of missed doses	
Dose 2	0/122 (0)
Dose 3^a^	2/109 (1.8)
Dose 4^b^	2/110 (1.8)
Dose 5^c^	3/100 (3.0)
Dose 6^d^	2/111 (1.8)

Action to be taken if tablets are vomited	
Go back to health facility for replacement dose	316 (57.3)
Give another dose	209 (37.8)
Do nothing	27 (4.9)
Don't know	0 (0)

With what was AL taken/given?	
Nothing	0 (0)
Water only	549 (99.4)
Food	2 (0.4)
Beverage	1 (0.2)
Other	0 (0)

Timing of tablet intake when administered with food?	
Before meal	171 (31.0)
During meal	2 (0.4)
After meal	379 (68.6)

What acted as a reminder to take tablets?^e^	
The dispenser's instructions	231 (41.8)
The pictograms	309 (55.9)
Illness	12 (2.2)
Other^f^	12 (2.2)

^a ^Dose used to replace vomited dose 2 (n = 1), dose not taken due to stomach ache (n = 1)

^b ^Dose delayed (n = 1), tablets lost (n = 1)

^c ^No reason given (n = 1), dose not taken due to excessive vomiting (n = 1), admitted to hospital due to asthma, treated with quinine (n = 1)

^d ^Forgot to take dose (n = 1)

^e ^Refers to morning dose (results were similar for evening dose)

^f ^Not specified

Nearly all patients took AL with water (549/552 [99.5%]). Two patients (0.4%) took the drug with food. The most frequently reported factor that positively influenced adherence to the timing of AL dosing was the impact of the current illness (Table 2).

### Acceptability of AL

Almost all patients (91.8%) found the dosing pictogram helpful, and all patients reported that clustering of tablets within the blister packs was useful (Table 3). In total, 87.1% of patients (481/552) found AL easier to take/administer than SP and 484/552 (87.7%) believed that AL was more effective than SP (Table 3). Approximately 90% of patients (495/552) would not have preferred other medications than AL to treat the current illness, although 5.6% (31 patients) would have chosen quinine injections. Two patients would have preferred to receive herbs from a traditional healer.

**Table 3:** Acceptability assessments (n = 552)

	N (%)
Were the instructions (drawings) in the AL pack useful?	
Yes	507 (91.8)
No	34 (6.2)
Don't know	11 (2.0)

Was the clustering of AL doses useful to remember how to take the drug?	
Helpful	552 (100)
Confusing	0 (0)
Not important	0 (0)

How do you or your child feel now?	
Very unwell	6 (1.1)
Unwell	5 (0.9)
Moderately unwell	3 (0.6)
Moderately well	47 (8.5)
Well	486 (88.0)
Perfectly well	9 (0.9)

How do you find AL to administer/take?	
Easier to take than SP	481 (87.1)
Less easy than SP	2 (0.4)
Same as SP	32 (5.8)
Don't know	37 (6.7)

Do you find that AL works?	
Yes	549 (99.5)
Better than SP	484 (87.7)
Same as SP	32 (5.8)
Don't know	35 (6.5)
No	3 (0.6)

Would you or your child prefer to have anything else than AL for this particular illness?	
No	495 (89.7)
Yes	57 (10.3)
No specific choice	21/57(36.8)
Antibiotics	1/57 (1.8)
Analgesics/antipyretics	2/57 (3.5)
Quinine injection	31/57 (54.4)
Herbs from traditional healer	2/57 (3.5)

## Discussion

Adherence to the standard AL regimen was very good in this population of patients in a rural area of sub-Saharan Africa. The full six-dose regimen was taken by 98% of patients, with the dose being taken at a satisfactory time in ~90% of cases. These results were obtained without any additional training for staff at the dispensing health center beyond the standard National Malaria Control Programme training initiative, and no special guidance was given to patients other than that which is routinely offered by the local healthcare personnel. The proportion of patients taking all five unsupervised doses was consistent with other reports for ACT in general [24,25] and AL in particular [26-29]. This is, however, the first study to provide data related to the timing of AL administration, and the first to apply a randomized study design to the assessment of AL adherence.

Although multiple doses of AL are required, there is no need for individual dose calculations according to body weight; instead, complete treatment packages are available for each body weight group. Thus, AL is simpler to prescribe than SP, the previous first-line therapy, which requires weight-adjusted dosing. However, patients need to take five doses of AL unsupervised over a three-day period compared to a single dose of SP. The findings, however, indicate that adherence to the AL regimen is very good following standard instructions from the dispensing healthcare provider, as confirmed by pill counts at the randomized visits. According to the patient responses to the questionnaire, the clustering of tablets for each dose within the AL blister packets is likely to have contributed to the correct number of tablets being taken, and the pictogram shown on the packaging was considered as a helpful supplement to the instructions provided by healthcare workers. The pictogram made the timing of drug administration clear in this rural area where few individuals have a clock or wrist watch [30]. Care should be taken, however, that dispensing staff explain that the 'sun' and 'moon' symbols refer to daytime and night-time, and not sunrise and sunset as believed in a few isolated cases among the study population. Finally, the wide-held response that AL is effective may also have played a role in supporting adherence to the regimen.

It was notable that responses indicated that AL was virtually never taken with food, despite the fact that health providers emphasized that AL works better if not taken on an empty stomach. Based on responses to the question about timing of tablet intake, it appears that some patients may have eaten shortly before or after taking the AL dose and did not consume additional food at the time of dosing, but specific information was not collected. It is encouraging, however, that a recent analysis of data from a large-scale study of AL in five African countries found that although concomitant food intake increased lumefantrine absorption in children with malaria, there was no tendency for lower food intake in the few patients in whom treatment failure was recorded [31]. Indeed, all 37 patients who were unable to eat food with any dose achieved PCR-corrected cure at day 28. Nevertheless, food consumption, or resuming food consumption as soon as possible, at the time of AL dosing remains advisable in order to maximize effectiveness, in view of the observed association between lumefantrine exposure and clinical and parasitological outcomes [23]. This can, of course, be challenging since initially patients may be reluctant to eat due to symptoms of nausea and vomiting during the acute phase of malaria.

Fewer than 60% of patients or caregivers understood the need to return to the health clinic for a replacement if a dose was vomited. Although the number of patients in whom a dose was reported to have been vomited was low (n = 2), the findings that more than 40% of patients or caretakers did not know what to do if this happens is of concern.

The current findings provide detailed evidence of the timely intake of AL under programmatic conditions. Other analyses of unsupervised adherence have all involved home visits after the three-day course was completed [26-29]. Encouragingly, however, no study has reported fewer than 90% of patients taking all six doses by the end of the three-day treatment period, based on pill counts. In this case, it was possible to validate oral information about pill administration in over 99% of cases, and found no difference between reported consumption and the remaining number of pills. Such validation is essential given the known limitations of self-reporting [32]. The findings of this study concur with those described recently by Bell *et al*, based on a study in Malawi in which children or adults with uncomplicated *P. falciparum *malaria were randomized to receive AL or chorproguanil-dapsone [29]. Of the patients randomized to receive AL, 100% reported correct pill consumption during oral interviews. However, in a subpopulation of 87 patients in whom pills were dispensed from an electronic pill container that recorded the time of opening, Bell *et al *found that the rate of adherence was only 92%. Thus, the adherence rate obtained using this different measurement approach was the same as that observed in this study population (92%), where we assessed adherence by randomized, scheduled visits shortly after each dose was due to be taken. Clinically, the high adherence rates observed when AL is self-administered after the first dose have been shown to result in excellent efficacy rates irrespective of whether the drug is given supervised or unsupervised [23].

Certain aspects of the study design merit consideration. The study was undertaken in the context of routine use of AL therapy in a rural area of sub-Saharan Africa. The healthcare providers who dispensed AL did not receive any special training in addition to the standard training provided through the National Malaria Control Programme, when first-line treatment with AL was introduced, approximately one year prior to this assessment. Also, the randomization approach helped ensure comparability of groups for each AL dose assessed, and minimized the influence of interviews by avoiding expected or repeated visits. However, we are aware that responses may be due to the phenomenon that patients or caregivers provided answers they thought would be expected or desired by the interviewer. Whether or not a pill had been removed from the blister pack could be checked through pill counts, but other questions - e.g. those in which the acceptability of AL was compared to other treatment options - could not be validated. Furthermore, responses for children under 13 years were given by parents or caregivers, not by the patient. Therefore, the views expressed in cases of children under 13 are those of their parents/guardians and may not necessarily reflect the patient's perspective. We also recognize that the questions posed could be refined, particularly those which resulted in 100% or near-100% responses, such as 'Was the clustering of AL doses useful to remember how to take the drug?'. Moreover, there is a need to standardize questionnaires that assess adherence and acceptability to improve the quality and subtlety of the information gained and to improve comparability between studies.

In conclusion, adherence to the AL regimen as standard first-line treatment of uncomplicated *P. falciparum *malaria was high among this rural study population. Patients adhered closely to the dosing regimen, partly due to effective packaging and pictorial dosing instructions and to patients' conviction that AL is effective. These results may be helpful for future training of healthcare providers by National Malaria Control Programmes in sub-Saharan Africa before and during implementation of ACT therapy as first-line anti-malarial treatment.

## Conflicts of interests

Funding for this study was provided from Novartis Pharma and Novartis Foundation for Sustainable Development. A M Kabanywanyi, B Genton and C Lengeler received honoraria and travel expense reimbursement from Novartis Pharma to present study findings at various international conferences. R Schlienger and N Mulure are employees of Novartis Pharma. P Kasim and S King'eng'ena have no conflicts of interest. A medical writer, funded by Novartis Pharma, provided editorial support based on the draft manuscript prepared by A M Kabanywanyi.

## Authors' contributions

AMK contributed to study design, was a study investigator and drafted the manuscript for input by the other authors. CL participated in questionnaire design, data interpretation and writing of the manuscript. PK and SK undertook data collection. RS contributed to study and questionnaire design and provided input to the manuscript. NM contributed to questionnaire design and acted as the medical advisor to the project. BG contributed to study and questionnaire design and provided input to the manuscript. All authors read and approved the final manuscript.

## Supplementary Material

Additional file 1 **Questionnaire**. COA566A2422/ALIVE: A community-based study to assess the impact of Coartem (ALu) when used as national policy first-line treatment on malaria mortality and morbidity in Tanzania Click here for file

## References

[B1] World Health Organization World Malaria Report 2008 WHO Press, World Health Organization, Geneva, Switzerland ISBN 978 92 4 156369 7

[B2] Rowe AK Rowe SY Snow RW Korenromp EL Schellenberg JR Stein C Nahlen BL Bryce J Black RE Steketee RW The burden of malaria mortality among African children in the year 2000 Int J Epidemiol 2006 35 691 704 10.1093/ije/dyl027 16507643 PMC3672849

[B3] Breman JG Alilio MS Mills A Conquering the intolerable burden of malaria: what's new, what's needed: a summary Am J Trop Med Hyg 71 Suppl 2 1S 15S 15331814

[B4] Roca-Feltrer A Carneiro I Armstrong Schellenberg JR Estimates of the burden of malaria morbidity in African in children under the age of 5 years Trop Med Int Health 2008 13 771 783 18363586 10.1111/j.1365-3156.2008.02076.x

[B5] Sachs J Malaney P The economic and social burden of malaria Nature 2002 415 680 685 10.1038/415680a 11832956

[B6] Ayieko P Akumu AO Griffiths UK English M The economic burden of inpatient paediatric care in Kenya: household and provider costs for treatment of pneumonia, malaria and meningitis Cost Effec Resour Alloc 2009 7 3 10.1186/1478-7547-7-3 PMC2640355 19161598

[B7] Djaman J Abouanou S Basco L Koné M [Limits of the efficacy of chloroquine and sulfadoxine-pyrimethamine in Northern Abidjan (Cote d'Ivoire): Combined in vivo and in vitro studies](in French) Sante 2004 14 205 209 15745869

[B8] Mugittu K Genton B Mshinda H Beck HP Molecular monitoring of Plasmodium falciparum resistance to artemisinin in Tanzania Malar J 2006 5 126 10.1186/1475-2875-5-126 17177997 PMC1764422

[B9] Mugittu K Abdulla S Falk N Masanja H Felger I Mshinda H Beck HP Genton B Efficacy of sulfadoxine-pyrimethamine in Tanzania after two years as first-line drug for uncomplicated malaria: assessment protocol and implication for treatment policy strategies Malar J 2005 4 55 10.1186/1475-2875-4-55 16297234 PMC1315332

[B10] Mugittu K Ndejembi M Malisa A Lemnge M Premji Z Mwita A Nkya W Kataraihya J Abdulla S Beck HP Mshinda H Therapeutic efficacy of sulfadoxine-pyrimethamine and prevalence of resistance markers in Tanzania prior to revision of malaria treatment policy: Plasmodium falciparum dihydrofolate reductase and dihydropteroate synthase mutations in monitoring in vivo resistance Am J Trop Med Hyg 2004 71 696 702 15642957

[B11] World Health Organization Guidelines for the treatment of malaria 2006 http://www.who.int/malaria/docs/TreatmentGuidelines2006.pdf Accessed 18 August, 2009

[B12] Kokwaro G Mwai L Nzila A Artemether/lumefantrine in the treatment of uncomplicated falciparum malaria Expert Opin Pharmacother 2007 8 75 94 10.1517/14656566.8.1.75 17163809

[B13] White NJ Antimalarial pharmacokinetics and treatment regimens Br J Clin Pharmacol 1992 34 1 10 1633062 10.1111/j.1365-2125.1992.tb04100.x PMC1381368

[B14] Falade C Makanga M Premji Z Ortmann CE Stockmeyer M de Palacios PI Efficacy and safety of artemether-lumefantrine (Coartem^®^) tablets (six-dose regimen) in African infants and children with acute, uncomplicated malaria Trans R Soc Trop Med Hyg 2005 99 459 467 10.1016/j.trstmh.2004.09.013 15837358

[B15] Faye B Ndiaye JL Ndiaye D Dieng Y Faye O Gaye O Efficacy and tolerability of four antimalarial combinations in the treatment of uncomplicated Plasmodium falciparum malaria in Senegal Malar J 2007 6 80 10.1186/1475-2875-6-80 17570848 PMC1919387

[B16] Fanello CI Karema C van Doren W Van Overmeir C Ngamije D D'Alessandro U A randomised trial to assess the safety and efficacy of artemether-lumefantrine (Coartem) for the treatment of uncomplicated Plasmodium falciparum malaria in Rwanda Trans R Soc Trop Med Hy 2007 101 344 350 10.1016/j.trstmh.2006.06.010 17005222

[B17] Omari AA Gamble C Garner P Artemether-lumefantrine (six-dose regimen) for treating uncomplicated falciparum malaria Cochrane Database Syst Rev 2005 4 CD005564 16235412 10.1002/14651858.CD005564 PMC6532733

[B18] Sinclair D Zani B Donegan S Olliaro P Garner P Artemisinin-based combination therapy for treating uncomplicated malaria Cochrane Database Syst Rev 2009 3 CD007483 19588433 10.1002/14651858.CD007483.pub2 PMC6532584

[B19] Coartem^®^/Riamet^® ^(artemether/lumefantrine) Basic Prescribing Information Novartis Pharma AG, Basel, Switzerland. Last updated September 2007 http://www.pharma.us.novartis.com/product/pi/pdf/coartem.pdf

[B20] Makanga M Premji Z Falade C Karbwang J Mueller EA Andriano K Hunt P De Palacios PI Efficacy and safety of the six-dose regimen of arthemeter-lumefrantrine in pediatrics with uncomplicated plasmodium falciparum malaria: a pooled analysis of individual patient data Am J Trop Med Hyg 2006 74 991 998 16760509

[B21] van Vugt M Looareesuwan S Wilairatana P McGready R Villegas L Gathmann I Mull R Brockman A White NJ Nosten F Artemether-lumefantrine for the treatment of multidrug-resistant falciparum malaria Trans R Soc Trop Med Hyg 2000 94 545 548 10.1016/S0035-9203(00)90082-8 11132386

[B22] Lefévre G Looareesuwan S Treeprasertsuk S Krudsood S Silachamroon U Gathmann I Mull R Bakshi R A clinical and pharmacokinetic trial of six doses of artemether-lumefantrine for multidrug-resistant *Plasmodium falciparum *malaria in Thailand Am J Trop Med Hyg 2001 64 247 256 11463111 10.4269/ajtmh.2001.64.247

[B23] Piola P Fogg C Bajunirwe F Biraro S Grandesso F Ruzagira E Babigumira J Kigozi I Kiguli J Kyomuhendo J Ferradini L Taylor W Checchi F Guthmann JP Supervised versus unsupervised intake of six-dose artemether-lumefantrine for treatment of acute, uncomplicated *Plasmodium falciparum *malaria in Mbarara, Uganda: a randomized trial Lancet 2005 365 1467 1473 10.1016/S0140-6736(05)66416-1 15850630

[B24] Ajayi IO Browne EN Garshong B Bateganya F Yusuf B Agyei-Baffour P Doamekpor L Balyeku A Munguti K Cousens S Pagnoni F Feasibility and acceptability of artemisinin-based combination therapy for the home management of malaria in four African sites Malar J 2008 7 6 10.1186/1475-2875-7-6 18182114 PMC2254635

[B25] Ajayi IO Falade CO Olley BO Yusuf B Gbotosho S Iyiola T Olaniyan O Happi C Munguti K Pagnoni F A qualitative study of the feasibility and community perception on the effectiveness of artemether-lumefantrine use in the context of home management of malaria in south-west Nigeria BMC Health Serv Res 2008 8 119 10.1186/1472-6963-8-119 18513447 PMC2429909

[B26] Chinbuah AM Gyapong JO Pagnoni F Wellington EK Gyapong M Feasibility and acceptability of the use of artemether-lumefantrine in the home management of uncomplicated malaria in children 6-59 months old in Ghana Trop Med Int Health 2006 11 1003 1016 10.1111/j.1365-3156.2006.01654.x 16827701

[B27] Rahman MM Donorp AM Day NP Lindegardh N Imwong M Faiz MA Bangali AM Kamal AT Karim J Kaewkungwal J Singhasivanon P Adherence and efficacy of supervised versus non-supervised treatment with artemether/lumefantrine for the treatment of uncomplicated Plasmodium falciparum malaria in Bangladesh: a randomised controlled trial Trans R Soc Trop Med Hyg 2008 102 861 867 10.1016/j.trstmh.2008.05.022 18606428

[B28] Fogg C Bajunirwe F Piola P Biraro S Checchi F Kiguli J Namiro P Musabe J Kyomugisha A Gushtmann J-P Adherence to a six-dose regimen of artemether-lumefantrine for treatment of uncomplicated *Plasmodium falciparum *malaria in Uganda Am J Trop Med 2004 71 525 530 15569777

[B29] Bell DJ Wootton D Mukaka M Montgomery J Kayange N Chimpeni P Hughes DA Molyneux ME Ward SA Winstanley PA Lalloo DG Measurement of adherence, drug concentrations and the effectiveness of artemether-lumefantrine, chlorproguanil-dapsone or sulphadoxine-pyrimethamine in the treatment of uncomplicated malaria in Malawi Malar J 2009 26 204 10.1186/1475-2875-8-204 PMC2744923 19709418

[B30] Dowse R Ehlers M Medicine labels incorporating pictograms: do they influence understanding and adherence? Patient Educ Couns 2005 58 63 70 10.1016/j.pec.2004.06.012 15950838

[B31] Borrmann S Sallas WM Marrast AC Kern SE Exposure to lumefantrine in infants and children receiving artemether-lumefantrine for uncomplicated malaria: impact of African diet components American Society of Tropical Medicine and Hygiene Annual Meeting, 7-11 December 2008, New Orleans, USA

[B32] Garber MC Nau DP Erickson SR Aikens JE Lawrence JB The concordance of self-report with other measures of medication adherence: a summary of the literature Med Care 2004 42 649 652 10.1097/01.mlr.0000129496.05898.02 15213489

