# Supplementary material for: Adherence to and acceptability of artemether-lumefantrine as first-line anti-malarial treatment: evidence from a rural community in Tanzania
Source: Malar J. 2010 Feb 11;9:48. doi: 10.1186/1475-2875-9-48 (PMC2833168; doi:10.1186/1475-2875-9-48)
Supplement: Additional file 1 — Questionnaire. COA566A2422/ALIVE: A community-based study to assess the impact of Coartem (ALu) when used as national policy first-line treatment on malaria mortality and morbidity in Tanzania [file 1475-2875-9-48-S1.DOC]

# COA566A2422/ ALIVE: A community-based study to assess the impact of Coartem (ALu) when used as national policy first-line treatment on malaria mortality and morbidity in Tanzania

# Adherence – feasibility study

| **I- Interview at the health facility:** | | | | | | | | | | |
| --- | --- | --- | --- | --- | --- | --- | --- | --- | --- | --- |
| **1-Name of the Health Facility:**  ________________________ | | **2-Name of Interviewer:**  _____________________ | | | **3-Patient number:**  |___|___|___| | | | **4- Date of the Interview:**  -----/---------/2008  *day/ month/ year* | | |
| **Patient demographic data** | | | | | | | | | | |
| **5-Name:_____________**  ________________________ | | **6-Sex:**   M   F | | | | **7-Age:**  Years: ……  Months: …… | | | **8-Regimen; total number of tablets:**  |___|___| | |
| **9-Village:**  ________________________  ________________________ | | **10-Hamlet (Kitongoji):** | | | | **12-House place/Neighborhoods:**  _____________________________ | | | | |
|  |  | **11-Hamlet leader/Famous person:**  _______________________ | | | |  |  |  |  |  |
| **Dose 1 administered at the health facility at Day 0** | | | | | | | | | | |
| **13-Date(treatment day 0)**  -----/---------/2008  *day/ month/ year* | | **14-Time of dose 1 administration**  -------:--------  *(24 hrs format)* | | | **15-Name of drug administrator:**  ________________________________ | | | | | |
| **II- Interview at the patient home:** | | | | | | | | | | |
| **1-Name of interviewer:**  __________________________________________ | | | | **2- Date of the interview:**  -----/---------/2008  *day/ month/ year* | | | | | | |
| **3- Was an informed consent obtained and signed?** Yes, No  *if yes please attach a copy to this questionnaire*  *if no please make sure consent is obtained and signed before the interview has started* . | | | | | | | | | | |
| **Visit details** | | | | | | | | | | |
| **4-Time of Visit:**  ……:…… **(24 hrs format)**   Morning  Evening   Midday  Night | | | | **5- Dose assessed:**   2^nd^ dose  3^rd^ dose  4^th^ dose   5^th^ dose  6^th^ dose   other, specify:_____________________________ | | | | | | |
| **Respondent details** | | | | | | | | | | |
| **6- Name of respondent/caretaker (in case of a child patient):**  ________________________ ________   Not applicable | | | **7- Relationship of the respondent to the child**   mother,  father   grandmother  grandfather   sister  brother   other specify:  _____________________________________________ | | | | | | | |
| **8- Level of education of caretaker/patient (in case of adult patient)**   None  Primary   Secondary  College | **9- Occupation of caretaker/patient (in case of adult patient)**   employed   self employed   farmer   other specify:_______________ | | | | | | **10- Age of respondent:**  …..........years | | | **11- Sex of respondent:**   M   F |
| **III-Adherence survey** | | | | | | | | | | |
| **1-** **When you went to the health facility, were you/your child?**   very unwell  unwell  moderately unwell   moderately well  well  perfectly well | | | | | | | | | | |
| **2-Where did you get the ALu medication for this sickness?**   Health facility,  pharmacy store,  street vendor,  ADDOs   other specify:……………………………………. | | | | | | | | | | |
| **3-How many tablets were in the ALu pack?**  5/6 10/12 15/18 20/24 (*tabs)* *(please circle the correct answer)* | | | | | | | | | | |
| **4-Did the health care provider or vendor explain to you how to administer ALu?**   Yes,  No,  don’t know | | | | | | | | | | |
| **5-Did the health care provider ask you whether you have understood how to administer ALu?**  Yes,  No,  don’t know | | | | | | | | | | |
| **6-(Excluding the first dose that was given at the health facility) how many doses in total will you administer for a complete course of treatment?**  1 2 3 4 5 6 *(please circle the correct answer)*   don’t know  other specify, ……………………………………………....................................... | | | | | | | | | | |
| **7-How many tablets per dose do you or does your child need to take?**   1 tablet  2 tablets  3 tablets  4 tablets  **please indicate if:**  Correct  Wrong  care-taker doesn’t know | | | | | | | | | | |
| **8-For this particular illness which dose did you last give/take?**  **(a)**  1^st^ dose  2^nd^ dose  3^rd^ dose  4^th^ dose  5^th^ dose  6^th^ dose  **(b) When was it?**  Time: ……..:…….. Date: ……../………/2008  *(24 hrs format) day/ month/ year*  **(c)** **Indicate if this is the same dose under assessment**   Yes,  No,  **(d) If No specify**_____________________________________________________________  **or**  **(d)** **If yes, please indicate the timing:**  Correct  Wrong | | | | | | | | | | |
| **9-Was the ALu pack available for the interviewer’s verification?**   Yes  No  **If yes**, count the remaining tablets,  **If not** explain.......................................................................................................................... | | | | | | | | | | |
| **10- How many tablets are still in the pack?***---------------------- (please insert number of tabs)* | | | | | | | | | | |
| **11-Was any dose missed?**   Yes  No **If yes** *please indicate and specify for each of the missing dose the reason.*  1 Reason_____________________________________________________  2 Reason_____________________________________________________  3 Reason_____________________________________________________  4 Reason_____________________________________________________  5 Reason_____________________________________________________  6 Reason_____________________________________________________ | | | | | | | | | | |
| **12**-**What would you do if you or your child throw(s) up/vomit(s) after taking the tablets?**   nothing  give another dose  go back to the health center  don’t know | | | | | | | | | | |
| **IV-Acceptability survey** | | | | | | | | | | |
| **1-Do you find the instructions (drawings) in the ALu pack useful?**   Yes  No, please specify……………………………………..........................  don’t know | | | | | | | | | | |
| **2-How do you find the clustered doses in the blister packaging for you to remember how to take the drug?**   helpful  confusing  not important | | | | | | | | | | |
| **3-How do you or your child feel now?**   very unwell  unwell  moderately unwell   moderately well  well  perfectly well | | | | | | | | | | |
| **4-How do you find ALu to administer/take?**   easier to take than SP  less easy than SP  same as SP  don’t know | | | | | | | | | | |
| **5-Do you find that ALu works?**  Yes No  **If no explain---------------------------------------------If Yes, please specify if ALu works:**   better than SP  less well than SP  same as SP  don’t know. | | | | | | | | | | |
| **6-Would you or for your child prefer to have anything else than ALu for this particular illness?** Yes No  **If Yes,** *please specify*   Antibiotics  Analgesics/antipyretics  quinine injection  herbs from traditional healer   remedy from witch doctor | | | | | | | | | | |
| **7-Did you/your child take another drug for that fever episode?**   Yes  No  **If yes please specify:**   Paracetamol (e.g. Panadol, Tylenol)   other, specify…………............................................................................................................... | | | | | | | | | | |
| **8-Did you go to the “traditional healer” too?**   Yes  No **If yes, please specify the reason:______________________** | | | | | | | | | | |
| **V- Feasibility survey** | | | | | | | | | | |
| **1-With what do you take/give ALu?**   Nothing  beverage,  water,  food,   other specify………………………………………................................................................................. | | | | | | | | | | |
| **2-With what did you/your child take the last dose of the drug?**   with beverage,  water only   with food  without anything  **If with beverage: which one(s) (specify……………………………………………………..).**  **If with food: which one(s) (specify……………………………………………………………).** | | | | | | | | | | |
| **3-When do you take the tablets if you take them with food?**   before  after  with the food | | | | | | | | | | |
| **4-(a) In the morning how do you remember that you should take the tablets**  the dispenser's instructions, the pictograms, illness,  other  specify_______________________________________________________________________  **(b) In the evening how do you remember that you should take the tablets**  the dispenser's instructions, the pictograms, illness,  other  specify**_____________________________________________________________** | | | | | | | | | | |
| **VI- Perceived adverse drug reactions** | | | | | | | | | | |
| **1-Did you observe something unusual after ALu intake?**   Yes  No  don’t know  **If ‘yes’ specify…………........................................................................................................** | | | | | | | | | | |
| **2-How long did the event last?**   hours**................**  days**...............** *(indicate the duration)* | | | | | | | | | | |
| **3-Was any medication taken for this event?**   Yes  No  don’t know  **If ‘yes’ specify…………........................................................................................................** | | | | | | | | | | |
| **4-Did ALu make you/your child sicker than he/she was?**   Yes  No  don’t know  **If ‘yes’ specify how?…………..............................................................................................** | | | | | | | | | | |
| **VII- For the Interviewer’ records** | | | | | | | | | | |
| **1-Was the adverse event reported to a health worker?**   Yes  No  don’t know | | | | | | | | | | |
| **2-Was an ADR form or an SAE form completed based on the event severity?**   Yes  No  don’t know  **if yes, please specify date of completion:**  ………/………./2008  *day/ month/ year*  **if no, please refer the patient to a health facility or contact Dr: …………………………..;** | | | | | | | | | | |
